# Image Sensors for Wave Monitoring in Shore Protection: Characterization through a Machine Learning Algorithm

**DOI:** 10.3390/s21124203

**Published:** 2021-06-18

**Authors:** Aimé Lay-Ekuakille, John Peter Djungha Okitadiowo, Diana Di Luccio, Maurizio Palmisano, Giorgio Budillon, Guido Benassai, Sabino Maggi

**Affiliations:** 1 Department of Innovation Engineering, University of Salento, 73100 Lecce, Italy; 2 Department of Computer Science, Technological University Bel Campus, 03 Kinshasa, Congo; johnpdjungha@unirc.it; 3 Science and Technologies Department, University of Naples “Parthenope”, 80133 Naples, Italy; diana.diluccio@uniparthenope.it (D.D.L.); giorgio.budillon@uniparthenope.it (G.B.); 4 CNR, National Research Council, Experimental Research Center, 82100 Benevento, Italy; maurizio.palmisano@cnr.it; 5 Engineering Department, University of Naples “Parthenope”, 80133 Naples, Italy; guido.benassai@uniparthenope.it; 6 CNR, National Research Council, Institute of Atmospheric Pollution Research, 70126 Bari, Italy; sabino.maggi@cnr.it; 7 Faculty of Engineering, International Telematic University UniNettuno, 00186 Rome, Italy

**Keywords:** image sensors, sensors and sensing systems, machine learning, real-time sensing for water waving, shore protection

## Abstract

Waves propagating on the water surface can be considered as propagating in a dispersive medium, where gravity and surface tension at the air–water interface act as restoring forces. The velocity at which energy is transported in water waves is defined by the group velocity. The paper reports the use of video-camera observations to study the impact of water waves on an urban shore. The video-monitoring system consists of two separate cameras equipped with progressive RGB CMOS sensors that allow 1080p HDTV video recording. The sensing system delivers video signals that are processed by a machine learning technique. The scope of the research is to identify features of water waves that cannot be normally observed. First, a conventional modelling was performed using data delivered by image sensors together with additional data such as temperature, and wind speed, measured with dedicated sensors. Stealth waves are detected, as are the inverting phenomena encompassed in waves. This latter phenomenon can be detected only through machine learning. This double approach allows us to prevent extreme events that can take place in offshore and onshore areas.

## 1. Introduction

The monitoring of sudden and extreme events [1] through the use of real-time sensors and sensing systems can be of paramount importance for setting up the actions necessary to prevent further risks and damages.

Sea motion involves a complex set of parameters, among which are the magnitude of longitudinal and vertical waves, sea temperature, wind, transport phenomena, coastal erosion [2]. In general, sea wave characterization can be performed by means of diverse techniques and instrumentation. Remote sensing is generally the common technique to measure sea parameters, especially those related to waves. Radar is the most important instrument for detecting surface wave properties, for instance in the X-band, hence radar reflectivity can display these properties [3]. However, airborne or satellite-based platforms display some limitations such as height and length sensitivity, and information acquisition by points instead of surface; this implies the need to extend the local punctual information to the whole adjacent area. Local measurement can be performed by specific instruments, notably floating accelerometer buoys [4] on water, altimeter gauges [5] connected to airplane operating with radio pulses, and other instruments working by using electrical and optical variations. The accelerometers allow the water displacement to be measured in function of time. These devices exhibit a limitation due to small wave displacements, i.e., less accuracy. The altimeters, instead, offer the advantage of being usable for large areas. In general, the instruments, working on electrical variations, leverage: (i) the resistive gauge to measure the water level thanks to a pair of wires of pre-established resistivity to be mounted in vertical position in the sea. The level of water varies the resistivity, hence, so does the voltage which is proportional to the level; (ii) the variation of capacitance is connected to the variation of the dielectric constant due to the presence of water. The capacitance is proportional to the water level. The above instruments suffer from dysfunctions due to marine organisms that usually cause fouling phenomena. Echo-sounder [6] can be a solution but it is not accurate during storms. Thus, onshore instruments with high penetrating radius in terms of vision could be a solution. That is the scope of this paper. Dedicated algorithms should be developed to extract features from vision sensors located onshore, i.e., on the coast. The main advantages are connected to the opportunity of extracting from the video cameras the same features that can be obtained with multi-parametric sensors, without problems related to the effects of storms on the sea surface. Another minor advantage of the proposed system is its low cost.

Waves propagating on the water surface [7] can be considered waves propagating in a dispersive medium, where gravity and surface tension at the air–water interface act as restoring forces. In shallow waters, i.e., when the wavelength λ≫d, where d is the water depth, the phase velocity $c_{p}$ is independent of the wavelength and can be written as

(1) $$c_{p}=\sqrt{gd}$$

where g is the acceleration by gravity, implying that waves in shallow waters do not exhibit frequency dispersion. Conversely, in deep waters, i.e., when λ/2<d, the phase velocity $c_{p}$ is independent of the water depth but is proportional to the square root of the wavelength λ:

(2) $$c_{p}=\sqrt{\frac{gh}{2\pi }}$$

Expressing the wavelength λ as a function of the wave frequency f, $\lambda =g/2\pi f^{2}$, we also obtain that in, deep waters, the phase velocity $c_{p}$ is inversely proportional to the frequency of the water waves. In intermediate water depths, the expression for the phase velocity becomes:

(3) $$c_{p}=\sqrt{\frac{gh}{2\pi \tanh(2\pi d/\lambda )}}$$

which reduces to Equations (1) or (2) in the limiting cases of shallow or deep waters, respectively. The velocity at which energy is transported in water waves is defined by the group velocity $c_{g}$. For shallow waters, there is no frequency dispersion and thus the group velocity is identical to the phase velocity, $c_{g}=c_{p}$, while for deep waters, the group velocity is always half the phase velocity, $c_{g}=c_{p}/2$. At intermediate water depths, the expression of the group velocity becomes more complicated:

(4) $$c_{g}=\frac{c_{p}}{2}\left(1+\frac{4\pi d/\lambda }{\sinh(4\pi d/\lambda )}\right)$$

however, it is basically equal to the phase velocity of deep waters with a correction factor that depends on the ration between the depth d and the wavelength λ.

Based upon these considerations [8], the work carried out in this paper aims to provide an up-to-date and comprehensive overview of the physical environment of the Bagnoli-Coroglio Bay.

The paper is organized as follows: the current section has presented the main motivation of the paper by illustrating the different techniques used in this work; Section 2 describes the local context where the activities are carried out; Section 3 deals with the imaging system, the pre-processing of the necessary information related to the different parameters to be used, included SAR observations, and the illustration of the machine learning approach that uses the cameras to make comparison with the previous pre-processing step according to two different algorithms; Section 4 depicts the results that cannot be directly produced by the current instrumentation, and lastly, the conclusions summarize the results and present an outlook of future work.

## 2. Study Area and Local Context

The industrial activity in Bagnoli, mainly focused on iron and steel manufacturing, began in 1904 and led to the construction of one of the largest steel centers in Europe, the ILVA steel plant of Bagnoli (later renamed as Italsider) which, at the peak of its growth, occupied an area of approximately 2 million km^2^. In 1930, the increasing demand for raw materials made it necessary to build huge infrastructures to allow the docking of large tonnage ships. Between 1962 and 1964, part of the marine surface was filled by Italsider, causing a serious macro-alteration of the natural coastline [9]. For over 80 years, this area was subjected to exploitation and strong industrialization processes focused on the production of steel, cement and fiber cement, suffering a degradation that continued throughout the last century. This degradation led to very high levels of pollution that affected the whole area, infiltrating the land, groundwater and marine area and causing a serious macro-alteration of the natural coastline, irremediably influencing its social and economic development as well as causing a serious macro-alteration of the natural coastline [10].

**Figure 1:**
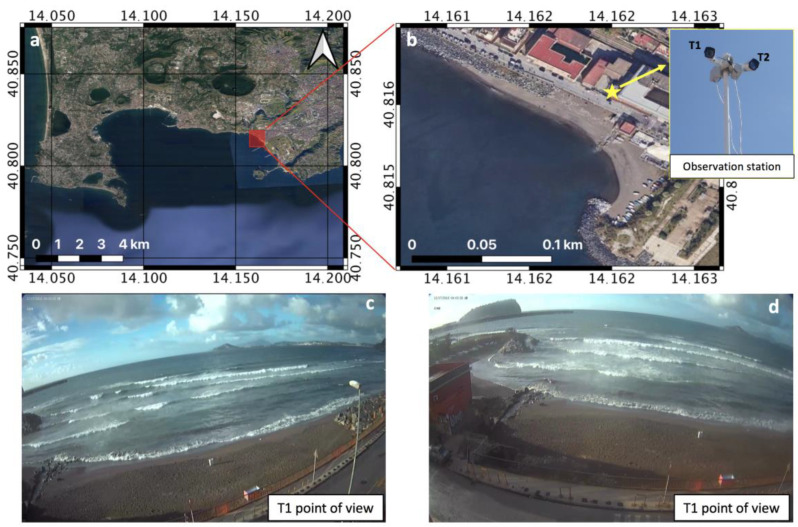
Overview of the study area (**a**) and zoomed-in image of the investigated beach (**b**), showing the points of view of the video-monitoring system (camera T1 (**c**) and camera T2 (**d**)).

The study area of this work includes a little beach located near the urbanized area of Bagnoli (Gulf of Napoli, southern Tyrrhenian Sea) (Figure 1). After 2000, the entire area was included among the Italian sites of high environmental risk, establishing the so-called “Sito di rilevante Interesse Nazionale” (site of relevant national interest) Bagnoli-Coroglio.

For over 80 years, this area was subjected to exploitation and strong industrialization processes focused on the production of steel, cement and fiber cement [11].

In 1990, the last industrial plant was also closed. Unfortunately, after so many years of intense industrial processes and activities, the damage was already done. The high levels of pollutants present in the soil and in the sea led to a severe program of redevelopment and the environmental remediation of the whole area. Today [12], the physiognomy of the area also appears deeply disturbed by indiscriminate urbanization processes that totally changed its profile and beaches, in addition to the effects caused by the industrial area, have undergone strong anthropic alterations. All these changes have contributed to modifying the marine currents in some sections of the study area, with a consequent effect on the coastline.

To characterize the environmental quality of the marine area impacted by the Bagnoli industrial site, provide an up-to-date and comprehensive overview of the physical environment of the Bagnoli-Coroglio Bay and develop a possible remediation and restoration strategy, extensive research projects have been carried out in recent years, as reported in [10].

## 3. Materials and Methods

Two main aspects are recalled in this paper, namely the sensors systems based on imaging and video-recording, and the algorithms used to process the output of the sensors.

### 3.1. Sensors Imaging

The video-camera observations were collected using a video-monitoring system installed in November 2018 in proximity of the test area (40°48′56.520″ N; 14°09′44.550″ E), as shown in Figure 1. The video-monitoring system consists of two separate cameras equipped with progressive RGB CMOS sensors that allow 1080p HDTV video recording [13]. The two cameras are oriented in the west–southwest (WSW) and south–southwest (SSW) directions and are mounted on a steel pole placed in front of the beach, at an elevation of approximately 30 m MSL (Figure 1b).

Every 30 min, all the images captured by each camera are saved in a different AVI file. The size of each 30 min file is approximately 0.5 GB. Since the AVI file size is limited to 2 GB for all system codecs, this design choice ensures the compatibility of the generated files with current tools and operating systems.

Images collected on the 16, 17 and 18 December 2018 from 07:00 UTC to 16:30 UTC with a 1 s time step were used for this work. The video camera measurements pointed out a deviation of the video-derived shoreline positions from those measured by GPS, correlated with the distance from a viewpoint and with the slope of the beach. In particular, the shoreline deviation of the T1 camera is approximately ±1 m and reaches a maximum of 1.8 m in the sectors where the beach curvature is larger, while the deviation of the T2 camera has a maximum value of 1 m.

### 3.2. Classical Numerical Method

To characterize the meteo-marine scenario during the video camera acquisition, a high spatial resolution model chain was configured using a High-Performance Computing (HPC) infrastructure that manages and runs the Weather Research and Forecasting (WRF), the Regional Ocean Model System (RMS3), and the WaveWatch III (WW3) open source model components [14].

**Figure 2:**
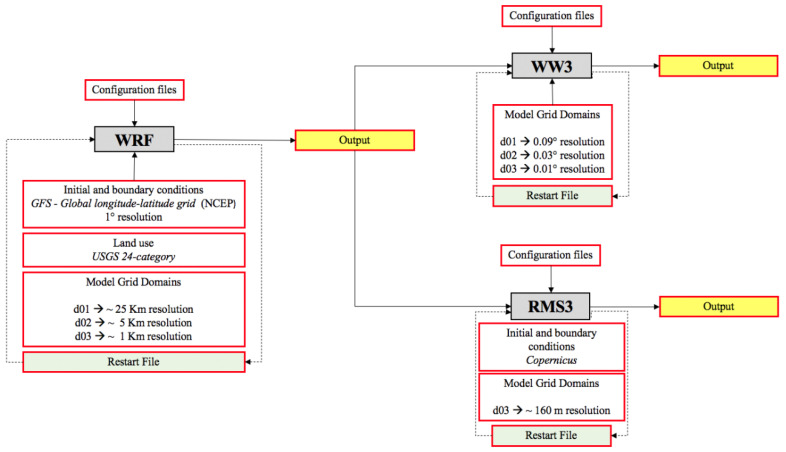
Block diagram of the model chain and of their mutual connections.

The model chain uses an offline coupling approach; the WRF atmospheric model component provides the hourly weather forecasts necessary as an initial condition for the WW3 offshore sea waves model component and for the hydrodynamic model RMS, as shown in the block diagram of Figure 2. To produce the numerical simulations of the 10 m wind fields presented in this paper, we configured the WRF model with two-way nested computational domains: a coarser domain (d01_WRF_) covering the whole of Europe and a finer domain (d02_WRF_) only covering the Italian peninsula, having a 25 and 5 km spatial resolution, respectively. The initial conditions of the WRF model were based on data from the Global Forecast System (GFS) produced by the National Center for Environmental Prediction (NCEP) [15].

Similarly, we configured three telescopic WW3 computing grids: the ground resolution on the whole Mediterranean area d01_WW3_ is 9 km, the resolution for the seas surrounding the Italian peninsula d02_WW3_ is set at 3 km, while the east sector of the central and southern Tyrrhenian Sea d03_WW3_ is covered at a resolution of 1 km [16].

The configuration of ROMS3 was provided using the sole d03_RMS3_ domain covering, at the spatial resolution of approximately 160 m, as the geographic area between the southern Lazio Region (Italy) and the northern Calabria Region (Italy) spanning for more than 100 × 50 nautical miles. Each of the configured models uses 1 h external time steps (with different internal time steps Δt for each model and domain) and support a restart mode (see Figure 2).

This wind numerical model configuration has already been used to support studies on coastal dynamics, coastal vulnerability assessment [17] and coastal management, as well as in combination with SAR-derived field observations.

### 3.3. Machine Learning-Based Approach

The movement of the waves can be considered as irrotational, and therefore, as the potential it derives from water is practically incompressible [18], this potential satisfies the Laplace equation. For wavelengths greater than 30 cm, surface tension can be neglected, and periodic solutions of low-amplitude air waves obey a dispersion relationship:

(5) $$\omega^{2}\;=\;g^{k}\tanh(kh)$$

with ω=2π/T: the pulse of the wave; T: the swell period; k=2π/L: wave number; L: the wave length of the swell; and h: the depth of the water. This relation gives the speed of propagation of the wave:

(6) $$c=\frac{\omega }{k}=\sqrt{\frac{g}{k}\tanh(kh)}$$

For the case of regular waves, this relation is correct at approximately 10% near in deep water, and the error can reach 30% in the case of shallow water as well. For this, the speed also increases with the amplitude of the waves. We note that the speed of the waves increases with the period, as the waves disperse, except in the limit of shallow depths. For this, the longest wave trains generated by a storm arrive before the shorter waves. For great depths, i.e., beyond half the wavelength, the speed of the waves no longer depends on the depth since the hyperbolic tangent tends towards 1.

The dispersion relation also makes it possible to understand the behavior of the waves [19] as they approach the coast. When the depth decreases spatially, the period remains constant. The above formulas lead to an increase in the number of wavelengths, and therefore a decrease in the wavelength and speed.

To simplify, in the case of deep water: velocity (propagation velocity or phase velocity) in m/s: $c=1.25\sqrt{L}$; period (time between two peaks): $T\approx 0.8\sqrt{L}$; and wave length: $L\approx 1.6T_{}^{2}$.

**Figure 3:**
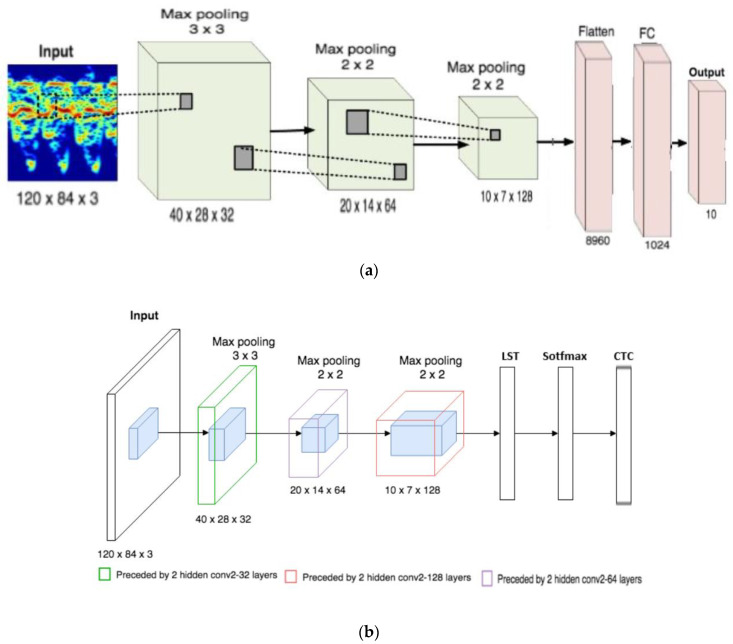
(**a**) Proposed double machine learning algorithm: a convolution including a multilayer perceptron (**left**); (**b**) Proposed double machine learning algorithm: 3D-CNN encompassing dedicated routines based on LSTM, Softm and CTC.

The aforementioned considerations are essential to implement the double algorithm illustrated in Figure 3a,b. Given the type of phenomenon and mechanism generating the envisaged outcomes, machine learning is the strong and right approach for extracting the desired data. We are not concerned with the distributions covering the epiphenomenon and whether they fall within, as for instance is the case of Lagrange’s, Poisson’s and Gauss’ descriptions, but we try to understand the global correlation amongst them. That is the main advantage of the machine learning approach. However, the inner arrangements of the algorithm are not obvious. These arrangements usually change according to the problem to be solved. Both Figure 3a,b are algorithm arrangements which are almost and slightly novel for the class of the problem we are facing in this paper. The first part, as per Figure 3a, is based on the acquisition of images captured by the video-sensors in order to retrieve the following data from the waves: temperature, vertical speed, and their magnitude. The algorithm is essentially based on convolution [20] along with a pooling of frames [21], and the final processing is provided by a multilayer perceptron (MLP) [22]. This first part must classify and extract features connected to temperature, vertical speed and wave detection. The convolution is devoted to applying the kernel, that is a few arrays of numbers, at the input to be connected to the output tensor, denoted as feature map, in this case maps of temperature, speed and wave detection. The pooling layer performs upon each feature of the above map in a separate way to create a new set of the same number of pooled feature maps. The pooling layer reduces the dimension of each feature map by a factor of 2.

The second algorithm, instead, extracts a volume of successive images to be, first processed by a 3D-CNN, and after through a cascaded LSTM (Long Short-Term Memory) [23], softmax [24], and a CTC block (connectionist temporal classification) [25]. In this case, the interest is in the composition of overlapped layers of waves, called superimposition, to retrieve and understand small particles of water contributing to the waving connected to the propagation speed.

Unlike RNN [26], the LSTM procedure provides small modifications to the information by multiplications and additions. The information is recorded by LSTM in the sense it selectively stores significant and useful parameters and forgets that is not useful. The chosen information is then processed by means of a softmax operator to maximize the magnitude of the previous information, also using an averaging operation. The last procedure is implemented by CTC that acts as a classifier that selects the most probable labeling related to the output of the softmax.

The aforementioned algorithms are connected to specific activation function, and considering the flowchart, we used the sotfmax as an activation function to solve the problem as well as max pooling layer blocks. The architecture we used has the same operating mode as that of the StoolNet of [27]. All convolutional layers are 1 “MP”, e.g., pooling layer, the pitches of all pooling layers are 3, and the core size is 3 × 3. “FC” stands for fully connected layer. The entry to StoolNet has been pre-processed, so that background information is discarded. The entry is resized to 120 × 84 × 3. The convolutional core sizes are 3 × 3 and the stride is 1. All activation functions in StoolNet are softmax.

The softmax function is also known to be used in various methods of classification into multiple classes, for example, in the case of artificial neural networks.

## 4. Results and Discussion

The test case was performed on Bagnoli beach, during the period 16–18 December 2018. The numerical model chain described in Section 3 was configured to reconstruct the weather and sea characteristics of the study area during this period.

**Figure 4:**
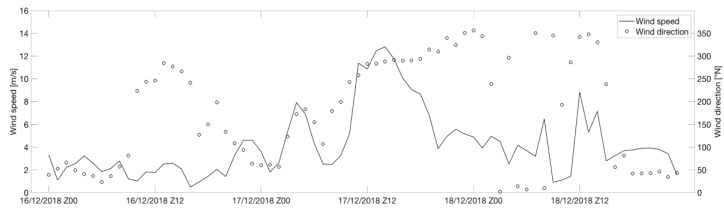
Calculation of wind speed (solid line) and wind direction (circles) in the study area between 16 and 18 December 2018. The simulation was performed using the WRF numerical model.

**Figure 5:**
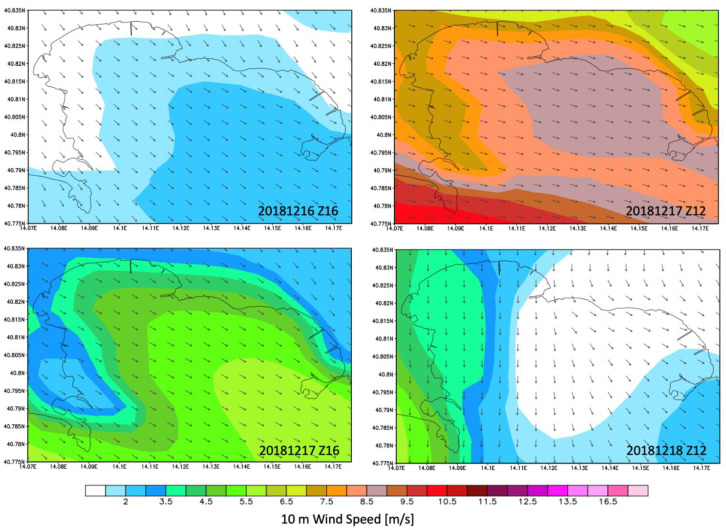
Maps of wind speed and wind direction in the study area between 16 and 18 December 2018.

As shown in Figure 4 and Figure 5, the study area was characterized by low winds coming from NW during the early hours of December 16 which underwent an hourly rotation in the following hours of the day, blowing from the NW at approximately 16:00 UTC. On 17 December 2018, the WRF meteorological model highlighted a maximum wind speed coming from NE with a maximum intensity of approximately 13 m/s at approximately 14:00 UTC. Subsequently, there was a decrease in wind speed in a range of 1–8 m/s mainly coming from N.

Starting from these weather forcings (wind), the numerical models WW3 and ROMS were used to perform the measurement of the sea state in terms of offshore waves [28], sea temperature, salinity and currents.

**Figure 6:**
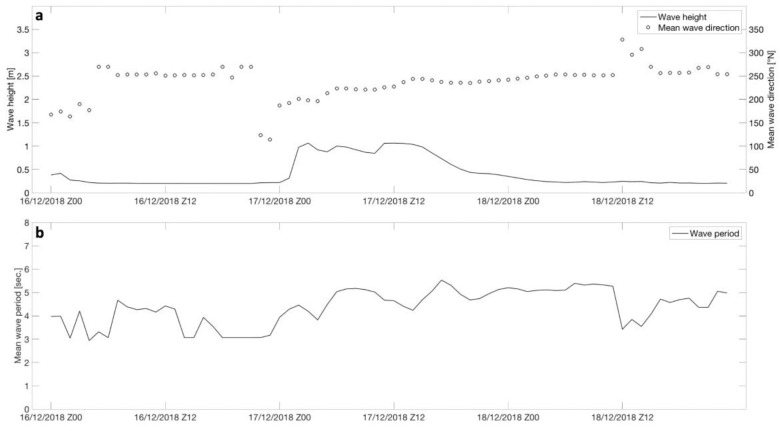
Calculation of wave height and direction (**a**) and wave period (**b**) in the study area between 16 and 18 December 2018. The simulation was performed using the WW3 numerical model.

As shown in Figure 6, the study area was characterized by the maximum significant wave height value (of approximately 1.3 m) on 17 December 2018, which is associated with an average period of approximately 5.5 s.

**Figure 7:**
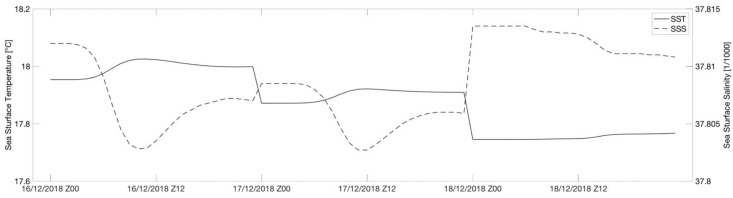
Calculation of sea surface temperature and salinity in the study area between 16 and 18 December 2018. The simulation was performed using the RMS3 numerical model.

The sea temperature and salinity define the weather column density, and indirectly, the vertical movements of the sea [29]. Figure 7 shows a sea surface temperature (SST) ranging from approximately 17.7 °C (during the night) and 18.1 °C. The sea surface salinity (SST) variations are very small.

**Figure 8:**
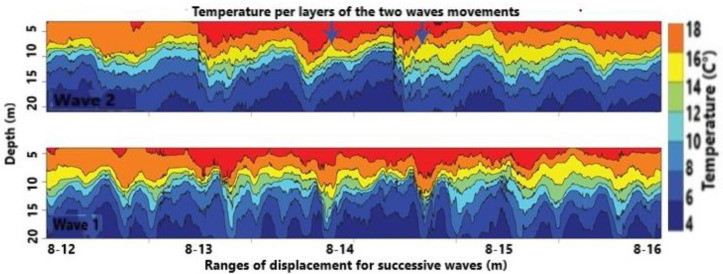
Temperature distribution during wave propagation and displacement steps.

The use of a machine learning approach reveals interesting features for decision-making allowing us to prevent damages. The adopted sensors, based on a progressive RGB CMOS device, allow 1080p HDTV video recording. The quality permits a good match for feature extraction based on artificial intelligence. As indicated in the previous section, using the first algorithm based on machine learning, we report an example of temperature distribution in December 2018 in function of the depth by considering two successive waves. The surface temperature, as per Figure 8, is approximately 10 °C for wave 1 (bottom band) in April 2019, and 18 °C for wave 2 (top band) in December 2018. The successive frames captured by the sensors are also reported. This is correct because, in December, in the area of the Naples gulf, the month of December is actually considered to be in autumn after a generally “strong” summer; conversely, April is in late mild winter.

**Figure 9:**
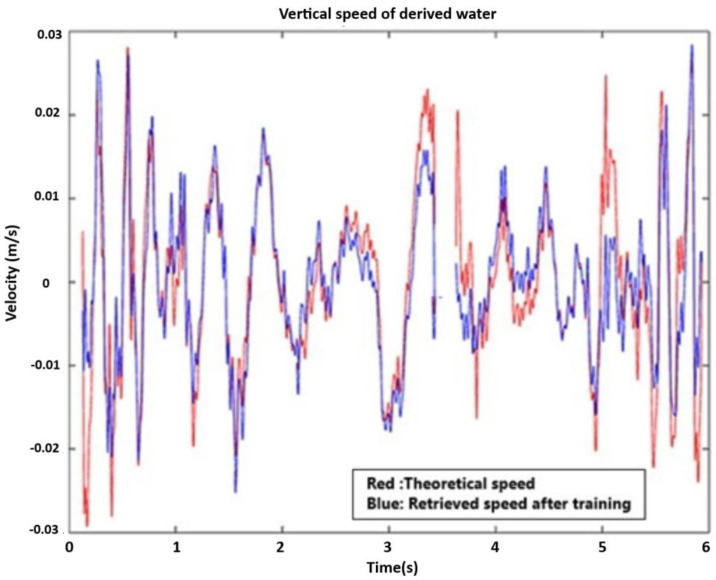
Vertical speed profile in theoretical and training steps.

**Figure 10:**
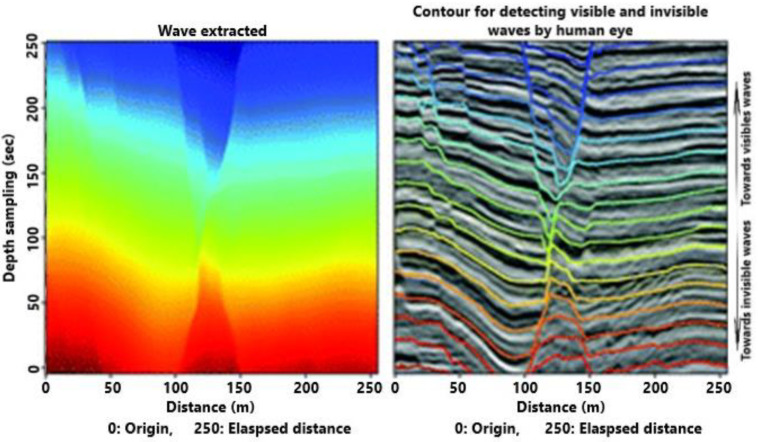
Wave field of propagation in frequencies visible and invisible to the human eye.

**Figure 11:**
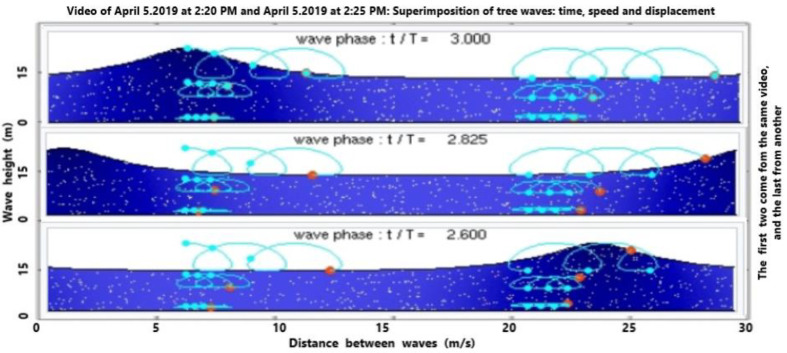
Output frames of processed overlapped wave propagation with 3D-CNN illustrating transport phenomena.

Figure 9 depicts the vertical speed of wave propagation with a hypothetical (theoretical) and processed profile recovered by the first algorithm. Certainly, there is a reduced magnitude in the middle area due to the evanescent effect connected to the overlapping of successive waves. This trend agrees with what is shown in the previous figure. To advance the content of the wave field of propagation, which is generally invisible to the human eye, thanks to the pooling procedure, we are able to distinguish with a preview the propagation of the wave field (Figure 10). This is very important in order to advance the useful actions before possible peaks. Now, we are able to detect the transport phenomena connected to partial irrotational wave propagation; that is, we can see a helicoidal motion of some particles (Figure 11). This aspect is very important, or instance, to detect fine solid and liquid materials with a different density than the sea water; for example, pollution due to oil spill.

The results need to be expressed with specific performance metrics. Two important indicators can be used, i.e., accuracy and the loss. Whilst the accuracy is well known, the loss is important in training algorithms. A loss function [30], also known as a cost function, takes into account the probabilities or uncertainty of a prediction based on the difference between the prediction and the true value. This gives us a more nuanced view of the model’s performance.

Unlike precision, the loss is not a percentage; it is a sum of errors made for each sample in training or validation sets, which is often used in the learning process to find the best values of parameters for the model with appropriate weights in the neural network. During the training process, the objective pursued is the only one to minimize or lower this value. The most common loss functions are log loss and cross entropy loss, which give the same result when calculating error rates between 0 and 1, as well as the root mean square error and probability loss. In contrast to the precision, the loss can be used in both classification and regression problems.

**Figure 12:**
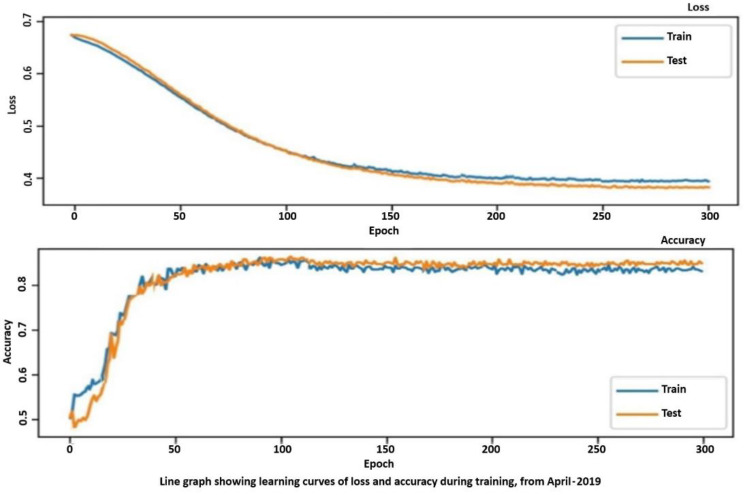
Accuracy vs. long epoch and loss illustration in April 2019.

**Figure 13:**
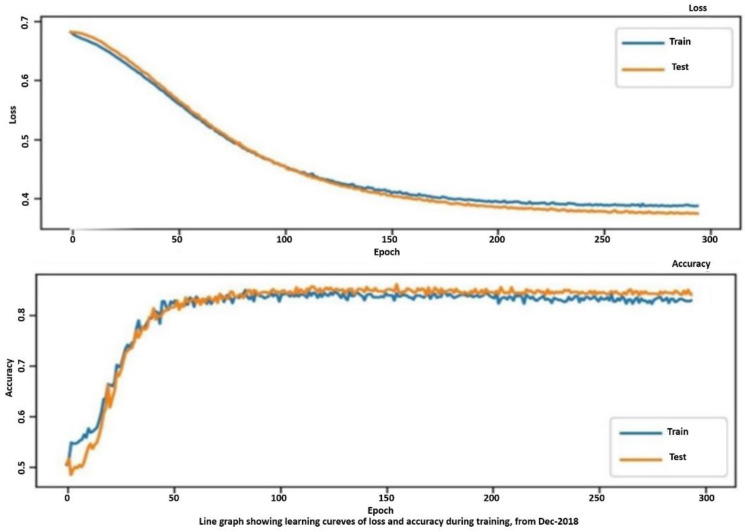
Accuracy vs. long epoch and loss illustration in December 2018.

**Figure 14:**
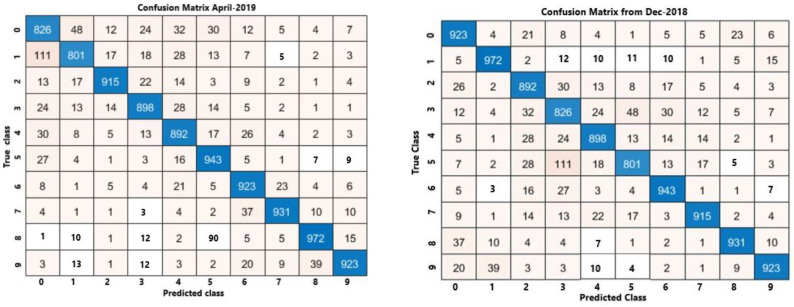
Confusion matrix metrics for April 2019 (**left**), and December 2018 (**right**).

The proposed metrics were applied to tailor the research in the effort to verify the training process related to the proposed algorithm. We recollect that two periods were involved. From Figure 12 and Figure 13, the trends of accuracy and loss are similar, respectively, for training (“train”) and delivered value (“val”), but there is a small discrepancy in the confusion matrix for both considered periods, i.e., April 2019 and December 2018. Since the trends are close, we introduced the confusion matrix as a further indicator to evaluate the eventual discrepancies. Figure 14 depicts both matrices for April (left) and December (right), whilst the value of the central diagonal are close, within a certain range of oscillation, but some values in the left area of the central diagonal indicate that there is a major discrepancy between the training and the delivered values for the data obtained in April. This “opening” or discrepancy starts after the ascending curve, after the 50th epoch and before the 100th epoch. It could be a question of wind and temperature distribution in two different periods: spring and autumn.

## 5. Conclusions

Certainly, the benefits of using sensors and sensing systems to extract features from a complex process involving different weather/climatic parameters is incommensurable. However, it is necessary to develop a robust algorithm to acquire these benefits in reference to the variability of the complex process. Video-sensors can assume a key role in recording the above complex process thanks to adequate post-processing. Extreme phenomena involving the sea and the ocean, in terms of marine waves, must be investigated, monitored in an online and offline manner. The latter requires recordings by means of sensors and sensing systems to understand the paroxysmal point that allows us to consider the phenomenon under test as reversible. That is, wave conditions along with wind fall in the range of the complex process.

The classical approach, in terms of modeling, delivers elements necessary for understanding such as wave magnitude, water surface temperature and wind fields. Certainly, in our case, an additional sensing instrument was necessary to retrieve local temperature and wind direction and intensity. However, these data can be given as input for a training algorithm based on artificial intelligence, namely machine learning to extract features encompassed in a panoply of data video-recorded by means of CMOS sensors. The classical approach, mostly deterministic and/or probabilistic, displays inaccuracy whether the set of data is not complete and/or in the presence of nonlinearities in the recovered data.

The current approach, also proposed in this paper, is based on collecting data from different sources such as weather forecast systems, and synthetic aperture radar (SAR) to be processed for retrieving accurate results. The cameras are used for counting the number of waves impacting on the shore. All these data are processed using a high spatial resolution model chain configured using a high-performance computing (HPC) infrastructure as depicted in Figure 2. It is a huge process with important deliveries in terms of results such as wind direction, magnitude, temperature and wave height.

Machine learning, instead, as was the case for us, enabled leveraging the information in order to produce useful features connected to parameters and variables which are hidden in the waves such as submarine temperature profiles, waves fields and transport phenomena. This approach is similar to virtual ultrasound monitoring [31]. Specific metrics have been adopted such as accuracy, loss and confusion matrix. They display reliable trends yielding to consider good training for the proposed algorithm.

Machine learning only works on the images captured by the video-sensors located on the shore. It delivers much more data than the previously illustrated model, that is: (i) temperature and wave range displacement in terms of successive waving. Numerically speaking, the results of temperature are very close, i.e., they display a difference less than 0.5–1.2%. Figure 8 exhibits a transitional layer from one pack to another with the relative temperature while the numerical model does not; (ii) the wave velocity is reported in both approaches. The training velocity is close to the theory envisaged for this area; (iii) the CNN allows to extract the wave field which is possible but difficult with the classical approach. Thanks to the wave field, we are able to extract the spatial wave displacement and impact in the visible and invisible range, as reported in Figure 10. This allows us to understand the submarine flow before the storm or in the absence of isokinetic conditions that complicate the application of classical models. The isokinetic conditions allow one to work in fluid equilibrium with a low Reynolds number; (iv) the 3-CNN, instead, delivers the transport phenomenon with the rolling and swelling as reported in Figure 11. The transport is certainly amplified by the wave height as mentioned previously. It is a paramount aspect for detecting material displacements, as in the case of oil spill due to discharges from vessels.

## Data Availability

Not applicable.
